# Analysis of the dynamic co-expression network of heart regeneration in the zebrafish

**DOI:** 10.1038/srep26822

**Published:** 2016-05-31

**Authors:** Sophie Rodius, Anna Fournier, Lou Götz, Robin Liechti, Isaac Crespo, Susanne Merz, Petr V. Nazarov, Niek de Klein, Céline Jeanty, Juan M. González-Rosa, Arnaud Muller, Francois Bernardin, Simone P. Niclou, Laurent Vallar, Nadia Mercader, Mark Ibberson, Ioannis Xenarios, Francisco Azuaje

**Affiliations:** 1 Oncology Department, NorLux Neuro-Oncology Laboratory, Luxembourg Institute of Health (LIH), Luxembourg, L-1526 Luxembourg; 2 Vital-IT Systems Biology Division, SIB Swiss Institute of Bioinformatics, Lausanne, CH-1015 Switzerland; 3 Oncology Department, Genomics Research Unit, LIH, L-1526 Luxembourg Luxembourg; 4 Vrije Universiteit Amsterdam, 1081 HV Amsterdam The Netherlands; 5 Cardiovascular Research Center, Massachusetts General Hospital and Harvard Medical School, Boston, MA 02114 USA; 6 Epicardium Development and Regeneration group, Centro Nacional de Investigaciones Cardiovasculares Carlos III (CNIC-ISCIII), 28029 Madrid Spain; 7 Center for Integrative Genomics, University of Lausanne, Lausanne, CH-1015 Switzerland; 8 Department of Biochemistry, University of Geneva, 1211 Geneva 4, Switzerland; 9 Present Address: Present address: Luxembourg Centre for Systems Biomedicine (LCSB), University of Luxembourg, Belvaux, L-4367, Luxembourg., ,; 10 Present Address: Present address: Department of Genetics, University of Groningen, Groningen, 9700 RB, The Netherlands., ,; 11 Present Address: Present address: Department of Development and Regeneration, Institute of Anatomy, Faculty of Medicine, University of Bern, Bern, Switzerland., ,

**Keywords:** Network topology, Transcriptomics, Regulatory networks

## Abstract

The zebrafish has the capacity to regenerate its heart after severe injury. While the function of a few genes during this process has been studied, we are far from fully understanding how genes interact to coordinate heart regeneration. To enable systematic insights into this phenomenon, we generated and integrated a dynamic co-expression network of heart regeneration in the zebrafish and linked systems-level properties to the underlying molecular events. Across multiple post-injury time points, the network displays topological attributes of biological relevance. We show that regeneration steps are mediated by modules of transcriptionally coordinated genes, and by genes acting as network hubs. We also established direct associations between hubs and validated drivers of heart regeneration with murine and human orthologs. The resulting models and interactive analysis tools are available at http://infused.vital-it.ch. Using a worked example, we demonstrate the usefulness of this unique open resource for hypothesis generation and *in silico* screening for genes involved in heart regeneration.

**Supplementary information:**

The online version of this article (doi:10.1038/srep26822) contains supplementary material, which is available to authorized users.

## Introduction

The adult zebrafish (*Danio rerio*) has the capability, unlike adult mammals, to fully regenerate its heart after substantial damage. This has been investigated with different *in vivo* models of cardiac injury^1^. The regeneration process depends on the dynamic interplay of multiple molecular components, which tightly control networks of transcriptional responses. A systematic understanding of the biological events that are required for heart regeneration in the zebrafish will open opportunities for discovering new therapeutic strategies for humans^2^. To enable novel insights with therapeutic potential, there is a need for modeling zebrafish heart regeneration through unbiased, systematic approaches.

Several studies have demonstrated that local ventricular injury triggers an organ-wide response that affects the major cardiac tissues and that subsequently promotes regeneration at the injury site^3,4,5,6,7^. Existing cardiomyocytes are the main source of new myocardium following ventricular injury^8,9^. Experiments suggest that during the regeneration process, cardiomyocytes need to reactivate a developmental program: transcription factors such as tbx5 and gata4 are re-expressed upon injury and inhibition of gata4 function impairs cardiac regeneration^10,11^. The reactivation of an embryonic program is consistent with the fact that regenerating cardiomyocytes acquire a phenotype specific to de-differentiated cells to facilitate cell division^6,8^. Epicardial cells highly contribute to ventricular regeneration by: (a) giving rise to both myofibroblasts and perivascular cells^12,13^, (b) promoting neovascularization^12,14,15^, (c) regulating extra-cellular matrix deposition in response to myocardial injury^13,16^, and (d) controlling cardiomyocyte migration to the wounded area as well as its proliferation^12,17,18^. MicroRNAs are well studied upstream regulators of many genes. Some miRNAs have recently shown to be regeneration regulators by directing cardiomyocyte proliferation and de-differentiation in the zebrafish, and represent promising targets with translational potential^19,20^.

In the past decade efforts have been made to understand the gene regulation underlying cardiac regeneration in the zebrafish. The first reports used microarrays to compare gene expression between controls and different stages post-amputation^21,22^. Such data were also compared to other regenerative models, such as tail regeneration, to identify processes specific to each tissue and those shared by different regenerative processes^23^. Changes in expression profiles of isolated cardiomyocytes have also been reported. Even though the latter yielded a reduced efficiency in the detection of differentially expressed genes, it was useful to determine cell type-specific gene regulation during cardiac regeneration upon genetic ablation of myocardium^24^. We recently analyzed some of the aforementioned datasets and predicted candidate genes with potential regulatory roles in heart regeneration^25^.

**Figure 1:**
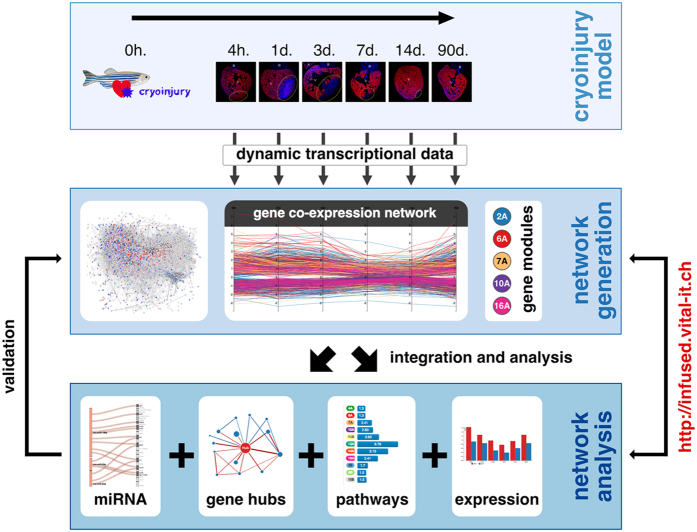
Discovery and resource development framework. Gene expression was measured and co-expression networks generated from control and injured zebrafish hearts at different times. Network structure analyses were implemented and their relations to biological function were determined, and top predictions were further investigated. This includes an *in silico* validation phase, which involved the establishment of association between the detected hubs (and modules) and independent external biological information from zebrafish and mammals (sources are indicated in box “network analysis”). A Web-based interactive resource is provided to enable the analysis, integration and visualization of these datasets. The zebrafish drawing was adapted from (http://bit.ly/1PL92vj) and is licensed under the Attribution-Share-Alike 3.0 Unported license (terms can be found at http://bit.ly/1pawxfE).

Here, based on data generated at our laboratory together with those publicly available, we substantially expand the knowledge gained from previous research by inferring and analyzing a dynamic gene co-expression network that underlies key stages of heart regeneration after cryoinjury in the zebrafish (Fig. 1). We identified network topology features that encode biologically-relevant properties. This network showed a highly modular organization that is linked to heart regeneration processes. We also found that highly connected genes within the network exhibit known, but also novel biological roles. We postulate that such hubs represent crucial transcriptional regulators of heart regeneration, and offer the community tools for hypothesis generation. Moreover, we demonstrate the potential functional importance of such hubs in different mammals. Our findings and online resource advance knowledge of the complex transcriptional web that elicits heart regeneration in the zebrafish, and will facilitate future research efforts.

## Results

### *In vivo* injury and regeneration of the zebrafish heart

**Figure 2:**
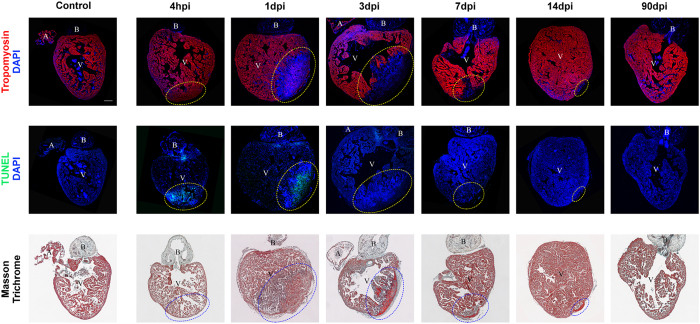
The different stages of heart regeneration in the zebrafish. Sagittal sections of adult zebrafish heart: anterior is towards the top and ventral towards the right. Hearts were cryoinjured and recovered at the indicated time points post-injury, and compared to healthy hearts from control fish. Sections were immunostained for tropomyosin in red, or TUNEL in green (nuclei are stained with DAPI in blue). Fibrosis was monitored by Masson-Goldner trichrome staining: healthy myocardial tissue (in red) and fibrotic areas (in blue). A: atrium; B: bulbus arteriosus; V: ventricle; hpi: hours post-injury; dpi: days post-injury. The injured area is indicated by a dotted circle. Scale bar is 100 μm.

Using a cryoinjury procedure^26^, we induced damage in adult zebrafish hearts. To dynamically monitor the regeneration process, we recovered samples at different post-injury times: 4 hours (hpi), 1, 3, 7, 14 and 90 days (dpi). Injured hearts were compared to healthy hearts from control fish in 3 independent experiments. We extracted RNA from heart ventricles for microarray experiments. We also recovered whole hearts to visualize healthy cardiomyocytes, apoptosis and fibrotic scar formation (Fig. 2). Healthy cardiomyocytes are observed in the whole ventricle and the atrium of control hearts, and injured hearts are totally devoid of such cells at 3 dpi. However, the size of the injury decreases all along the regeneration process, while new healthy cardiomyocytes are added from the border zone of the injured region, until 90 dpi when there are no visible (anatomical/cellular) differences with controls. Whereas massive cell death (in green, Fig. 2) is visible at the injury site as soon as 4 hpi and until 1 dpi, control hearts lack apoptotic cells. Lastly, while blood accumulates in the infarcted area at 1 dpi, formation of the fibrotic scar (in blue) is observed in the injury site at 3 dpi. Scar size decreases during the regeneration process and the injured area is gradually occupied by newly formed cardiomyocytes. At 90 dpi, ventricles are indistinguishable from controls. These results are concordant with previous work^5,25^ and corroborate the relevance of our model.

### Time-specific changes of gene expression during heart regeneration

**Figure 3:**
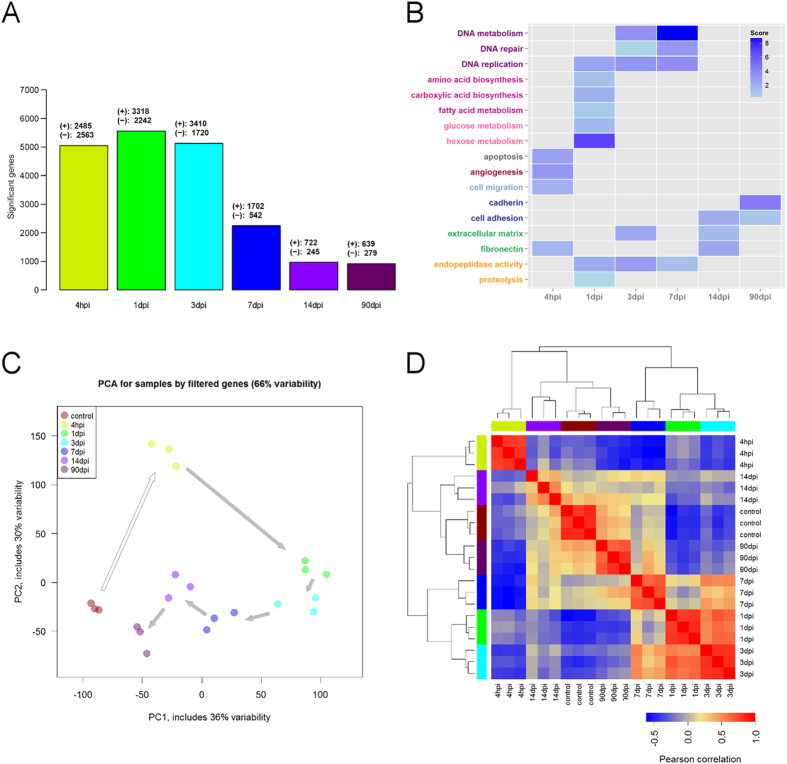
Significant changes in gene expression during heart regeneration. (**A**) Numbers of DEGs at each time point. (**B**) Summary of statistically enriched functional terms of DEGs at each time point. (**C**) Visualization of control and post-injury samples based on PCA (genes with FDR < 0.001). (**D**) Hierarchical clustering of samples based on gene expression data (genes with FDR < 0.001).

Using whole-genome microarrays, we obtained expression profiles at 4 hpi, 1, 3, 7, 14 and 90 dpi. Independent measurements were obtained in triplicates at each time point, and from 3 control samples. The differential expression of genes at each time in relation to controls was statistically estimated. The largest numbers of differentially-expressed genes (DEGs, FDR < 0.001) were obtained at the early stages of regeneration (from 4 hpi to 3 dpi). Although at later times the number of DEGs progressively decreased, thousands of statistically detectable changes were still observed (FDR < 0.001, Fig. 3A). The DEGs obtained at each time are enriched in functional annotations relevant to heart regeneration, such as apoptosis and angiogenesis (Fig. 3B, Supplementary Table S1). As early as 4 hpi, enrichments in genes involved in apoptosis (consistent with our staining, Fig. 2), angiogenesis and cell migration are statistically detectable. Previous work by our group has also reported angiogenesis from 3 days post-injury onwards. The expression of angiogenesis-involved genes precedes the development of new vessels. The fact that this activation can occur early cannot be explained solely by the time needed for gene transcription and translation. Notably, many angiogenic marker genes are also expressed in circulating hematopoietic cells. This is the case, for example for fli1a, kdlr or erg^27,28^. Given the fact that the infiltration of inflammatory cells is an early response to injury, it is possible that hematopoietic cells expressing endothelial cell markers can also home to the injured hearts.

At 1 dpi, transcriptomic alterations mostly impact genes implicated in energy metabolism, amino-acid biosynthesis and DNA replication, which could be an indication of enhanced cell proliferation. Changes are also observed in genes involved in proteolytic activities, indicating the beginning of the regeneration process^29,30^. At 3 and 7 dpi, the regeneration activity is boosted, as shown by the enrichment in peptidase activity, together with processes linked to cell proliferation such as DNA metabolism and replication. Also the extracellular matrix is highly implicated at 3 dpi, when fibrosis becomes visible in our histology staining. Processes related to cell adhesion are mainly enriched at later times. Altogether these results reflect crucial steps of the heart regeneration process.

By applying principal component analysis (PCA) to our gene expression data (DEGs, FDR < 0.001), it was possible to visualize well-defined time-specific clusters that clearly mirrored the ordered sequence of regeneration events (Fig. 3C). A hierarchical clustering of the data also distinctly separated samples according to expression patterns and times (Fig. 3D). These results indicate that our gene expression data reflects biologically-meaningful dynamic changes associated with heart regeneration.

### A co-expression network characterizes dynamic heart regeneration states

To characterize heart regeneration at a systems-level, we focused on significant transcriptional changes that distinguish injured from healthier hearts. This analysis was also needed to reduce the large number of expressed genes and facilitate network generation and analysis. First, we identified DEGs in the “injured group” (samples at 4 hpi, 1, 3, 7 and 14 dpi) vs. those in the “healthier group” (control and 90 dpi regenerated hearts). Because 90 dpi samples are anatomically very similar to healthy hearts, and at 90 dpi the hearts are actually at the end stages of full heart regeneration, their assignment to the “healthier group” is both reasonable and supported by our analysis. This procedure and the processing of genes with multiple probes resulted in a set of 3467 genes (FDR < 0.005) that were considered for subsequent analyses. Note that, although overlaps are observed, this set of genes differs from those represented in Fig. 3A, which only correspond to DEGs in the specific times vs. controls.

Next, we computed the expression (Pearson) correlations between these genes, and focused on gene associations with relatively high (absolute) correlation coefficients, │*r*│>0.8. This filtering was necessary to reduce potential spurious associations and to focus on those correlations likely to be biologically informative. This selection allowed us to achieve a balance between network scale-free fit and connectivity properties as recommended previously^31^. Moreover, this choice made subsequent network visualization interpretable and annotation tasks manageable.

The combination of all the selected gene-gene associations resulted in a global co-expression network of heart regeneration. The network consists of 3467 genes (nodes) and 436,803 associations (edges). Color-coding of the nodes on the basis of their expression fold-changes (in relation to controls) gives an overall view of the dynamic changes of the network at different times (Supplementary Fig. S1). This highlights systems-level response patterns that underlie the regeneration process: from prominent changes at the early stages of regeneration to more subtle, fine-tuned responses at later stages. It also allows us to appreciate the gradual regression from massive heart damage to a network state that resembles that exhibited by control samples (Supplementary Movie S1).

### Network modularity is linked to heart regeneration responses

**Figure 4:**
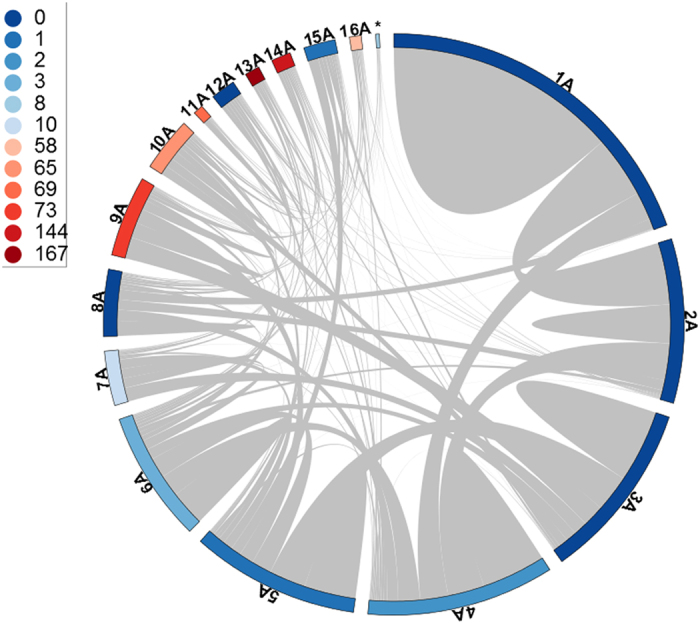
Modular architecture of the gene co-expression network in the zebrafish heart regeneration. Circular plots of modules detected with WGCNA: Internal links (grey) represent the intra- and inter-module connectivity, whereas the color of the outer bar represents the number of functional terms significantly enriched in a given module (Fig. 5). Numbers shown next to the colored circles (left panel) indicate the number of functional terms associated with each color in the plot.

**Figure 5:**
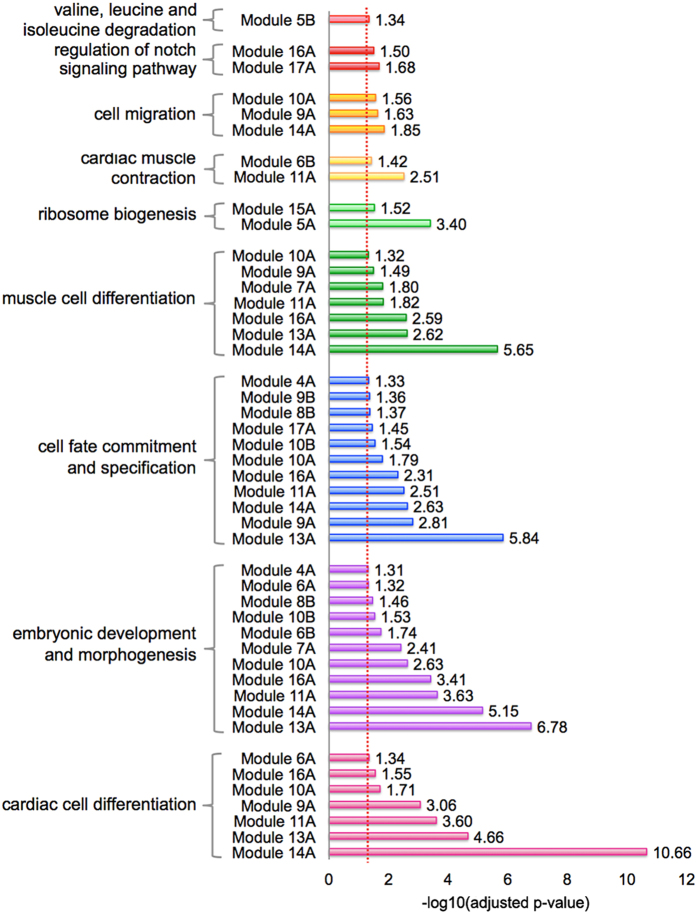
Summary of functional enrichments of modules. Dotted line represents threshold of statistical significance at FDR = 0.05 (Supplementary Methods).

Biological networks can be organized into modules of highly interconnected genes, which are implicated in the same biological processes or associated with specialized functions^32^. Here we identified modules of highly co-expressed genes through the application of network clustering algorithms. We focused on two techniques with well-established module detection capacity: WGCNA (Weighted Gene Co-Expression Network Analysis)^33^ and ClusterONE (Clustering with Overlapping Neighborhood Expansion)^34^. These techniques have enabled several biological network investigations, and represent complementary approaches to detecting network modules. We applied WGCNA to the global co-expression network and detected 17 modules (modules 1A to 17A), with sizes ranging from 38 (module 17A) to 707 genes (module 1A). With ClusterONE we identified 11 statistically significant modules (modules 1B to 11B), with sizes ranging from 8 (module 11B) to 491 genes (module 1B). A pairwise comparison of WGCNA and ClusterONE modules corroborated that the two techniques offer partially overlapping, complementary views of modularity (Supplementary Fig. S2). The largest overlap is represented by modules 11A (WGCNA) and 6B (ClusterONE) with Jaccard similarity coefficient of 0.706 (Supplementary Fig. S2). Apart from the expected high intra-module co-expression, there is a diversity of strong inter-module associations (Fig. 4, online resource). Moreover, a GO enrichment analysis shows that many of these modules (12 WGCNA and 4 ClusterONE modules) are significantly associated with biological processes relevant to heart regeneration (FDR < 0.05) (Figs 4 and S3, numbers of associations indicated in color legend). This includes cardiac cell differentiation, migration and embryonic development (Fig. 5).

To further determine the biological meaning of these results, we looked deeper into the gene composition and connectivity of these modules. Here we concentrate on module 14A because it is highly interconnected with other modules (Fig. 4) and consists of 61 highly co-expressed genes. This module also includes several genes with multiple connections with genes in other modules (Supplementary Fig. S4), and displays one of the largest numbers of statistically enriched biological processes implicated in heart regeneration, including muscle cell differentiation and cardiac cell differentiation (Fig. 5). It is highly enriched in processes related to embryogenesis, such as “embryonic development and morphogenesis” and “cell fate commitment and specification”. This indicates that genes in module 14A may play critical regeneration regulatory roles, promoting reactivation of the embryonic program which leads to dedifferentiation and further cell proliferation and differentiation (Fig. 5). Different genes found in Module 14A, such as *nkx2.5* and *csrp1*, are indeed key regulators of cardiac or vascular embryogenesis. The transcription factor *nkx2.5* (Supplementary Fig. S4) initiates the cardiogenic differentiation program in zebrafish^35^. This gene is a marker of cardiac progenitor cells and is re-expressed at the resection plane following ventricular amputation in the adult zebrafish^14^. Likewise *csrp1*, a member of the Wnt pathway, coordinates cardiac mesoderm cell migration during zebrafish embryonic development and its inactivation leads to cardiac bifida^36^.

Other genes such as *tbx5* and *ctgf*, may also be relevant to cardiac regeneration. Indeed, *tbx5*, a transcription factor naturally expressed in the developing heart, is required (together with *gata4* and *mef2c*) to allow reprogramming of cardiac fibroblasts into cardiomyocytes^37^. Whereas *ctgf* is a regulator of fibrosis during maladaptive remodeling in humans^38^. Other genes in module 14A have not yet been implicated in cardiac regeneration. They include *dyrk2*, *LOC100535315* (Supplementary Fig. S4), and *zgc:110366* which is still uncharacterized. The *dyrk2* kinase negatively regulates cardiomyocyte growth^39^, and thus may play an important role in the restoration of the correct organ size during regeneration. Future research will be required to validate the relevance of such genes in cardiac regeneration.

### Network hubs play controlling roles in heart regeneration

The identification of network hubs is useful to understand network function. Hubs may represent genes with influential biological roles or regulatory activity^32^. In the case of gene co-expression networks, hubs are genes exhibiting a statistically significant number of strong connections with other genes in the network. We applied the WiPer technique^40^ to identify hubs in our gene co-expression network of heart regeneration, and identified 425 genes that display a statistically-detectable large number of strong connections (adjusted-P < 0.05). Examination of top hubs located in different network modules highlighted the diversity of molecular functions potentially triggered or mediated by these genes (online resource). *Il6st* (also known as *gp130* receptor), which promotes the differentiation of embryonic stem cells into cardiomyocytes via the *gp130*/*jak2*/*stat3* pathway^41^, is overexpressed in proliferating cardiomyocytes following ventricular amputation^24^. Moreover, impaired regulation of *il6st* promotes adverse remodeling following myocardial infarction (MI)^42^. The disintegrin and metalloproteinase *adam8* mediates cell adhesion, migration, signaling and angiogenesis via proteolysis of various substrates^43^. Interestingly, *adam8* improves muscle fiber regeneration by regulating inflammatory reactions that are necessary to eliminate injured fibers prior to the regeneration step^44^, and single nucleotide polymorphisms in this gene are associated with MI^45^. Among other top-ranked hubs, *stx11a* and *cd63* are markers of intracellular vesicles, while *arpc5a* and *cotl1* are actin-binding proteins that may modulate cell migration and immune response during heart regeneration^46,47^. Other top-ranked hub genes are regulators of the inflammatory response, such as: *mrc1b*, *tmem154*, *igsf6* and *cd22*, which corroborates the importance of the immune response in heart regeneration (online resource). Lastly, other hubs (such as *si:ch211-264f5.2*) are still uncharacterized and represent interesting candidates for future investigation.

### Hubs are relevant to heart regeneration in mammals

We investigated the biological importance of the network hubs in different mammals with limited, but inducible, heart regeneration capacity. First, we determined the levels of homology of the hubs in mouse, rat and human. We found that a large majority of hubs have orthologs in human (78% of hubs), mouse (79%) and rat (78%) (Supplementary Table S2). Furthermore, hubs are statistically enriched in evolutionary conserved genes in comparison to other (non-hub) genes in our network. We detected this significant association in humans (P = 0.02, ^2^ = 5.67, Chi-square test), mouse (P = 0.0001, ^2^ = 15.15) and rat (P = 0.0003, ^2^ = 13.18).

Among our hubs, there are genes with mouse homologous whose importance in neonatal heart regeneration have been previously reported. Some of such hubs (LOC100331505, csf2rb, max, rb1, epas1a) mapped to DEGs or their putative regulators in a recent heart regeneration study by O’Meara *et al.*^48^. Others (zgc:123190, zgc:77517, ctssb.1) have homologous genes that are DEGs in fully regenerated hearts in mice as reported by Haubner *et al.*^49^.

**Figure 6:**
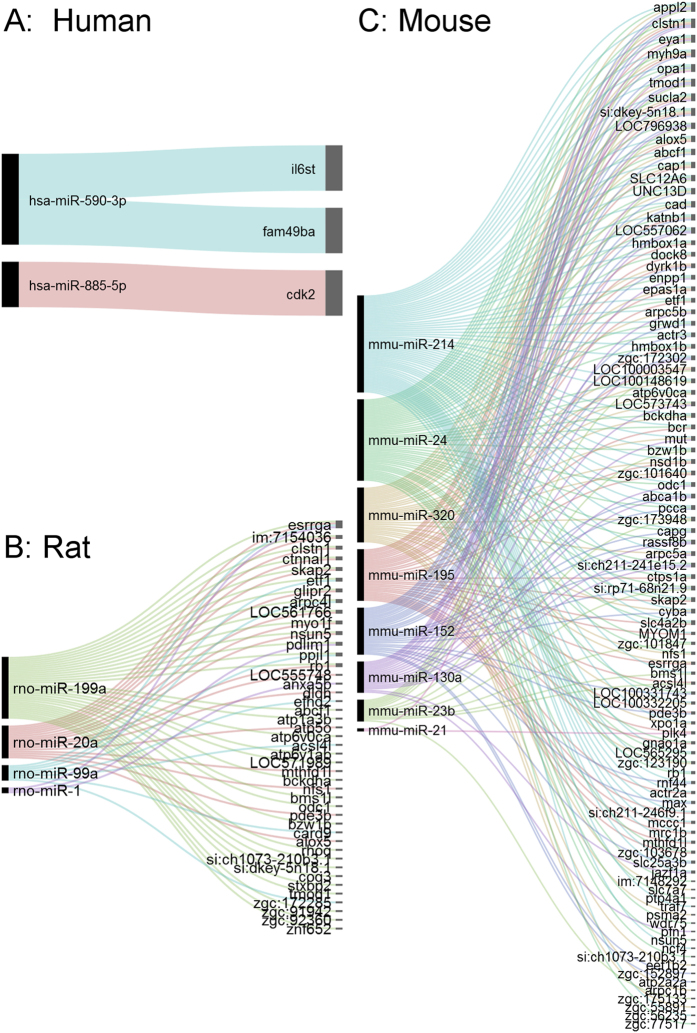
Network hubs are functionally important for heart regeneration in mammals. It offers an overall view of biologically important associations between hubs and miRs known to be drivers of regeneration in mammals. Higher resolution views, including their integration with other biological information, are provided on the website. (**A**) miR-hub interactions in humans. (**B**) miR-hub interactions in the rat. (**C**) miR-hub interactions in the mouse. Lines are used to indicate miR-target interactions, and are colored to group miR-specific interactions.

Next, we investigated whether the homologous genes are implicated in regulatory processes that are crucial for heart regeneration in mammals. We analyzed regulatory relationships between our hubs and 20 microRNAs (miRs) whose capacity to induce heart regeneration in mammals has been previously demonstrated^19,50^. Specifically, we addressed the question of whether our hubs are potential targets of miRs that are known to function as regeneration drivers. This was done by first generating a list of experimentally-validated miRs and their interactions in mouse, rat and human. Also we gathered putative miR-target interactions predicted by multiple computational techniques. We then searched for orthologs of our network hubs in the resulting datasets (Fig. 6). In humans, two of our hubs (*fam49ba* and *il6st*) are known targets of hsa-miR-590-3p, which has the capacity to trigger cardiomyocyte proliferation in (neonatal and adult) mice and rats^50^. We also identified 18 (unique) computationally-inferred interactions between our hubs and other heart regeneration miRs: hsa-miR-1, hsa-miR-195 and hsa-miR-199a^51^. In mouse and rat, we did not detect experimentally-validated interactions between regeneration miRs and hub orthologs, but we found hundreds of computationally-inferred hits. The latter included not only putative interactions with the mammalian heart regeneration miRs, but also with other miRs known to be relevant to cardiac cell proliferation and differentiation in mammals (Fig. 6).

A closer look at these interactions showed that, for instance, miR-199a targets the homologs of *esrrga* and *rb1*. *Esrrg* is highly expressed in the heart at fetal and postnatal stages, where it coordinates the oxidative metabolic program^52^. This gene is crucial for promoting the reprogramming of fibroblasts into cells of the cardiac lineage^53^. *Rb1* plays a fundamental role in priming embryonic stem cells toward cardiac cells^54^. As in the case of miR-199a, miR-195 also targets *esrrg*. Moreover, mirR-195 targets *alox5* and *epas1a*. The human homolog of *alox5* is vital to improve healing after MI through the regulation of inflammation and collagen production^55^. *Epas1* promotes angiogenesis and may support the adaptation of cardiomyocytes to hypoxia during heart failure^56^. Thus, our analyses show that mammalian homologs of network hubs are targeted by functionally-relevant miRs, which strengthens the biological importance of our predictions. Furthermore, our approach expands knowledge of miR-target associations that may be relevant to understand, and possibly elicit, heart regeneration in mammals.

### A web resource enables mining of key cardiac regeneration genes in zebrafish

**Figure 7:**
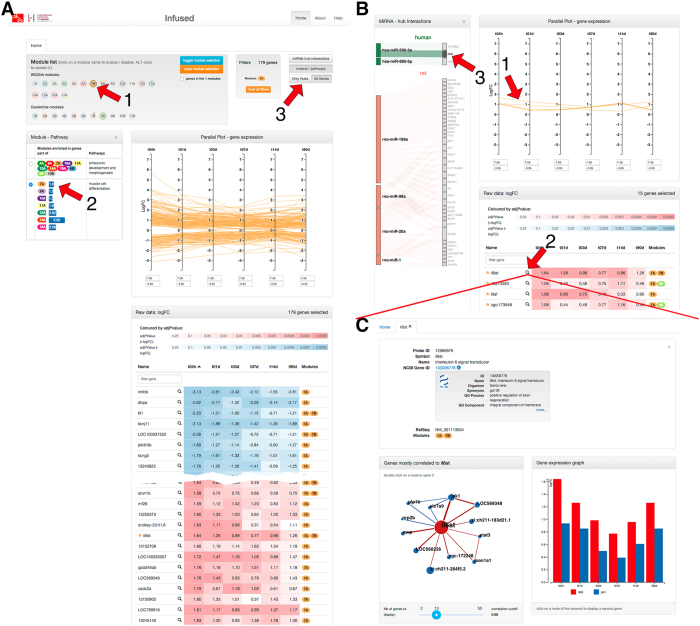
The web platform enables to explore the resource content; example of *il6st*. Select a module to explore (**A**-**1**, module 7A). Parallel coordinate plots as well as colored table of gene expression values (log_2_ fold change) present the profile and significance of gene expression changes in response to cryoinjury. Hub genes are marked with a star in the table. The functional categories enriched in this module are listed in (**A**-**2**). Panel (B) presents a restricted view on hub genes (available by clicking on **A**-**3**). (**B**-**3**) reveals a putative link between *il6st* and miR-885-5p in human. A detailed view on *il6st* gene (**C**) is displayed after clicking on (**B**-**2**). A summary of the NCBI gene record of that gene is available. (**C**-**1**) displays the network of genes most correlated to *il6st*. By clicking on any of the nodes (*Jak1* in this example), the expression fold change of the gene is displayed side-by-side against *il6st* (**C**-**2**).

We integrated the time-course differential expression, module/hub analysis and microRNA data into a self-contained Web resource enabling exploration of our data through an intuitive interface. Figure 7 illustrates how the interface can be used to identify a potential key gene and associated correlation network involved in the regeneration process. Since each module represents a set of correlated genes modulated during the cardiac regeneration process, we suggest using the modules as a starting point for investigating gene signatures. In the example shown in Fig. 7, module 7A, containing 179 genes, is first selected (Fig. 7A-1). Through the interface we can see that this module, along with several other modules, is significantly enriched for genes involved in embryonic development and muscle cell differentiation (Fig. 7A-2). We can select to show only genes that are “Hub” nodes according to our analysis (Fig. 7A-3); this highlights 15 genes. Further selection based on differential expression at early time-points (Fig. 7B-1) identifies *Il6st* as a “hub” gene that is up-regulated early on in the cardiac regeneration process (from 4 hpi) (Fig. 7A-2). The network of genes most correlated with *il6st* can be explored and the expression fold changes of the genes viewed side-by-side against *il6st* (Fig. 7C). This reveals a number of highly correlated genes including Jak1, which is the tyrosine kinase responsible for transducing the signal from the *Il6st* receptor complex, and *Stat3*, a transcriptional co-activator of the signaling cascade. Inspection of potential mammalian microRNAs targeting *il6st* indicates that *hsa-miR-590-3p* (Fig. 7B-3), which has been previously shown to impact cardiac regeneration, potentially regulates this gene at the post-transcriptional level. This is just one example of how new hypotheses on pathways involved in the regeneration process can start to be built rapidly using our Web resource. A more detailed step-by-step example is available on the website (help section).

## Discussion

We systematically investigated heart regeneration in the zebrafish in the context of gene co-expression networks. Our study is the first to provide such a systems-level characterization. Apart from enabling global, integrative insights of major transcriptional changes, our investigation identified significant associations between key topological properties of such networks and biological functions that are crucial for heart regeneration. We found that the regeneration process is mediated by different modules of highly co-expressed genes, which are jointly and dynamically implicated in processes relevant to major hallmarks of zebrafish heart regeneration. As the annotation of the zebrafish genome progresses, clearer and more diverse functional associations are likely to be detected. Our investigation also identified central genes with strong connectivity patterns in the network. Such hubs include genes with known or suspected roles in heart regeneration, as well as others whose novelty warrants future investigations. We demonstrated that hubs are significantly enriched in genes with mammalian orthologs, including those mapped to human. Furthermore, we identified functional relationships between our hubs and miRs that are known to induce heart regeneration in mammals. In the long-term, the induction of heart regeneration following MI in humans may be feasible through the dynamic targeting of genes (e.g., hubs) or sub-networks (e.g., modules) that are functionally conserved (and actionable) in the zebrafish and humans. Particularly, as cardiac regeneration in neonatal mouse models and also in humans has been reported^49,57^.

Although the biological importance and potential translational value of these findings will require further investigations, our study enables the zebrafish and heart regeneration research communities to integrate and analyze multiple datasets at the systems and cross-species levels. Our Web-based resource has the potential to facilitate a deeper understanding of heart regeneration in the zebrafish and accelerate the translation of this knowledge into therapeutic applications in humans.

## Methods

An overview of methods follows, and further details are available in the Supplementary Methods.

### Animal experiments and histological staining

Experiments on zebrafish conformed to regulatory standards and were approved by the Animal Welfare Structure of Luxembourg. Cryoinjury was performed as described in^26^. The methods were carried out in accordance with the approved guidelines and regulations. Hearts were immunostained for tropomyosin or processed for TUNEL staining as reported in^25^.

### Transcriptome profiling assays

For each time point and control, five cardiac ventricles per biological replicate were pooled in TRIzol® (Invitrogen, Carlsbad, CA). Extraction was performed as described in^25^. Transcriptome profiling assays were performed using Zebrafish GeneChip 1.0 ST arrays (Affymetrix, Santa Clara, CA). Data are available at the NCBI’s GEO database (Accession Number: GSE67665).

### Gene expression data analysis

Microarray data were pre-processed with Partek® Genomics Suite (v6.5) using the robust multi-chip analysis (RMA)^58^. We used empirical Bayes method from *limma* package of R/Bioconductor for differential expression analysis as in^59^. Functional enrichments of DEGs were analyzed with DAVID^60^.

### Gene co-expression network generation

Preprocessed microarray data were filtered by variance paired with a FDR method. To construct the weighted co-expression network, we applied the WGCNA^33^. Briefly, we calculated Pearson’s correlations between the probes and converted the correlation matrix into a weighted adjacency matrix with threshold β = 6. This resulted in a network that exhibited a good balance between: scale-free fit, median connectivity values and modularity. Also we analyzed other topological properties such as density and heterogeneity. To facilitate visualization and reduce potential spurious correlations, we filtered out edges with weights below 0.26, corresponding to Pearson’s correlation < 0.8.

### Analysis of network modules and hubs

Modules were detected with WGCNA^33^ and ClusterONE^34^. We applied WiPer^40^ to identify hub genes based on their (weighted) connectivity scores. Genes with statistically detectable connectivity (adjusted-P < 0.05) were defined as hubs.

### Gene orthology analysis in mammals

The zebrafish symbols were mapped to human, mouse and rat NCBI gene IDs using four different methods: retrieval of homolog gene IDs from The Zebrafish Model Organism Database (ZFIN)^61^ or GeneCards^62^, Homologene searches^63^, and BLAST searches for those genes without hits in these databases.

### Hubs as miR targets in zebrafish and mammals

We investigated the involvement of miRNAs in the regulation of the hubs previously identified by querying the miRTarBase resource^64^, an experimentally-validated microRNA-target interactions database. Also we harvested predicted microRNA-target interactions by querying the miRNAMap database^51^. We also explored the possible regulation of the hubs by miRNAs with a known role in cardiac regeneration, angiogenesis, fibrosis and apoptosis in other species (human, mouse and rat).

## Additional Information

**How to cite this article**: Rodius, S. *et al.* Analysis of the dynamic co-expression network of heart regeneration in the zebrafish. *Sci. Rep.* **6**, 26822; doi: 10.1038/srep26822 (2016).

### Supplementary information

Supplementary Table S1 (PDF 655 kb)

Supplementary Information (XLS 69 kb)

Supplementary Movie S1 (GIF 5561 kb)
