# Supplementary material for: Analysis of the dynamic co-expression network of heart regeneration in the zebrafish
Source: Sci Rep. 2016 May 31;6:26822. doi: 10.1038/srep26822 (PMC4886216; doi:10.1038/srep26822)
Supplement: Supplementary file 1 — Supplementary Table S1 (PDF 655 kb) [file 41598_2016_BFsrep26822_MOESM17_ESM.pdf]

Supplementary Information

Analysis of the dynamic co-expression network of heart regeneration in the zebrafish

Sophie Rodius [1], Anna Fournier [1,2], Lou Götz [3,12], Robin Liechti [3,12], Isaac Crespo [3], Susanne Merz [1], Petr V. Nazarov [4], Niek de Klein [1,5,6], Céline Jeanty [1], Juan M. González-Rosa [7], Arnaud Muller [4], Francois Bernardin [4], Simone P. Niclou [1], Laurent Vallar [4], Nadia Mercader [8,9], Mark Ibberson [3], Ioannis Xenarios [3,10,11,*], Francisco Azuaje [1,*].

[1] NorLux Neuro-Oncology Laboratory, Oncology Department, Luxembourg Institute of Health (LIH), Luxembourg, L-1526, Luxembourg.

[2] Current affiliation: Luxembourg Centre for Systems Biomedicine (LCSB), University of Luxembourg, Belvaux, L-4367, Luxembourg.

[3] Vital-IT Systems Biology Division, SIB Swiss Institute of Bioinformatics, Lausanne, CH-1015, Switzerland.

[4] Genomics Research Unit, Oncology Department, LIH, Luxembourg, L-1526, Luxembourg.

[5] Vrije Universiteit Amsterdam, Amsterdam, 1081 HV, The Netherlands.

[6] Current affiliation: Department of Genetics, University of Groningen, Groningen, 9700 RB, The Netherlands.

[7] Cardiovascular Research Center, Massachusetts General Hospital and Harvard Medical School, Boston, MA 02114, USA.

[8] Epicardium Development and Regeneration group, Centro Nacional de Investigaciones Cardiovasculares Carlos III (CNIC), Madrid, 28029, Spain.

[9] Current affiliation: Department of Development and Regeneration, Institute of Anatomy, Faculty of Medicine, University of Bern, Bern, Switzerland.

[10] Center for Integrative Genomics, University of Lausanne, Lausanne, CH-1015, Switzerland.

[11] Department of Biochemistry, University of Geneva, 1211 Geneva 4, Switzerland.

[12] These authors contributed equally.

* Corresponding authors: ioannis.xenarios@isb-sib.ch (I.X.), francisco.azuaje@lih.lu (F.A.)

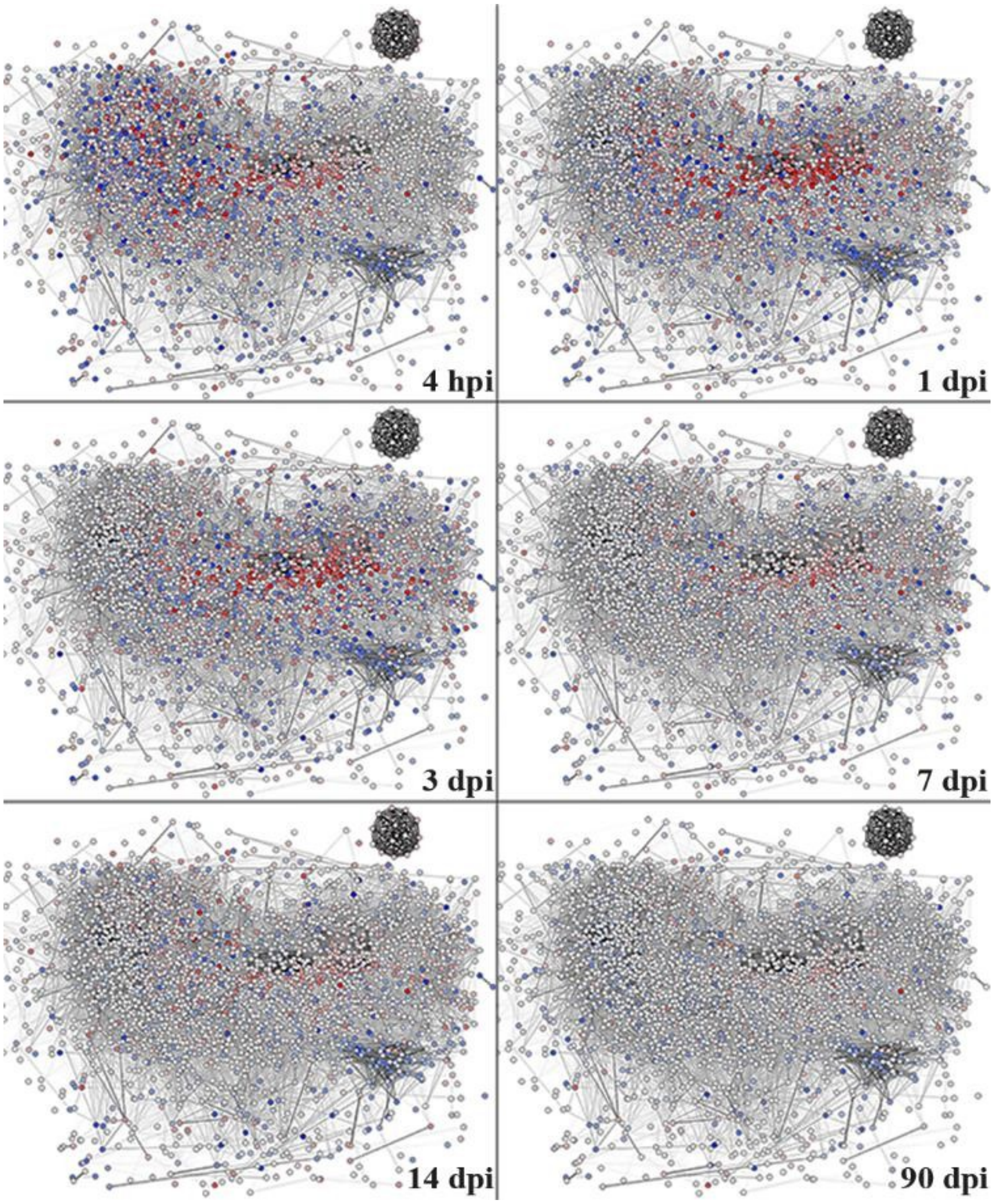

Figure S1. Dynamic, Network-Level Changes in Gene Expression during Heart Regeneration, Related to Figure 3. Global view of changes in gene expression (fold-changes in relation to controls) is provided for each heart regeneration time point. Nodes represent genes, edges represent expression correlations. Red nodes: Up-regulated, blue: Down-regulated, white: no change.

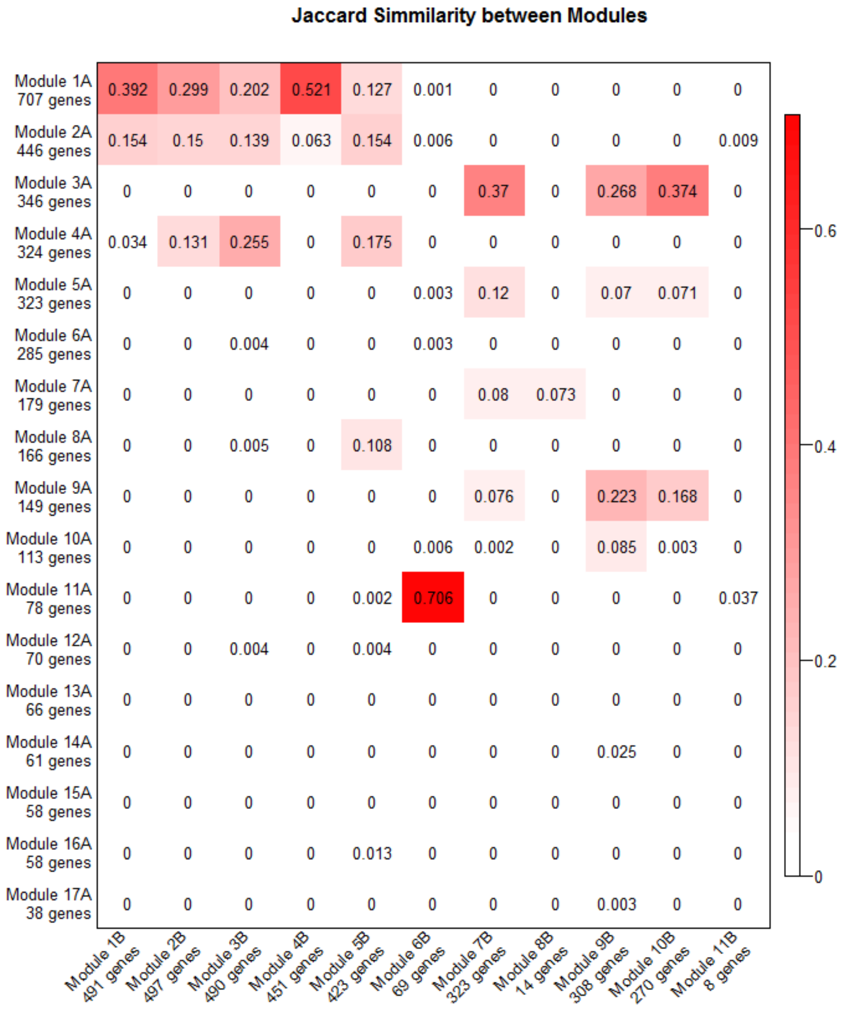

Figure S2. Overlaps Between Networks Modules Detected by WGCNA and ClusterONE, Related To Methods and Results (Network Connectivity Is Related to Heart Regeneration Features). Rows/columns represent modules detected by each method, cells represent the level of overlap between two modules. Module overlap quantified with the Jaccard similarity coefficient, with values from 0 (no overlap, in white) to 1 (perfect overlap, in red). The Jaccard similarity index is the ratio of the size of the overlap to the size of the union of two modules. Jaccard indices were computed with the made4 package (Culhane et al., 2005).

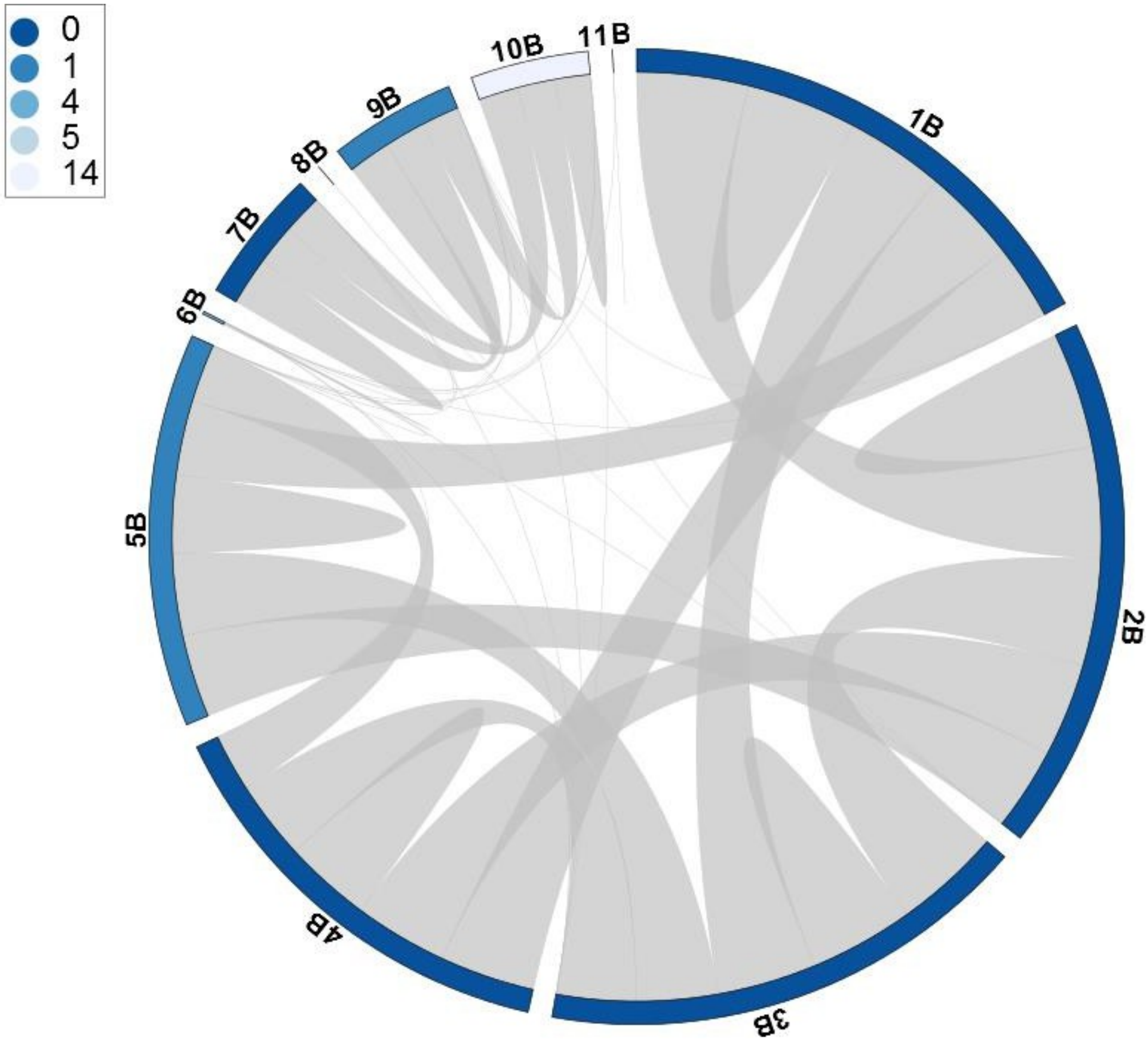

Figure S3. Modular architecture of the gene co-expression network in the zebrafish heart regeneration. Circular plots of ClusterONE modules: Internal links (grey) represent the intra- and inter-module connectivity, whereas the color of the outer bar represents the number of functional terms significantly enriched in a given module (Figure 5).

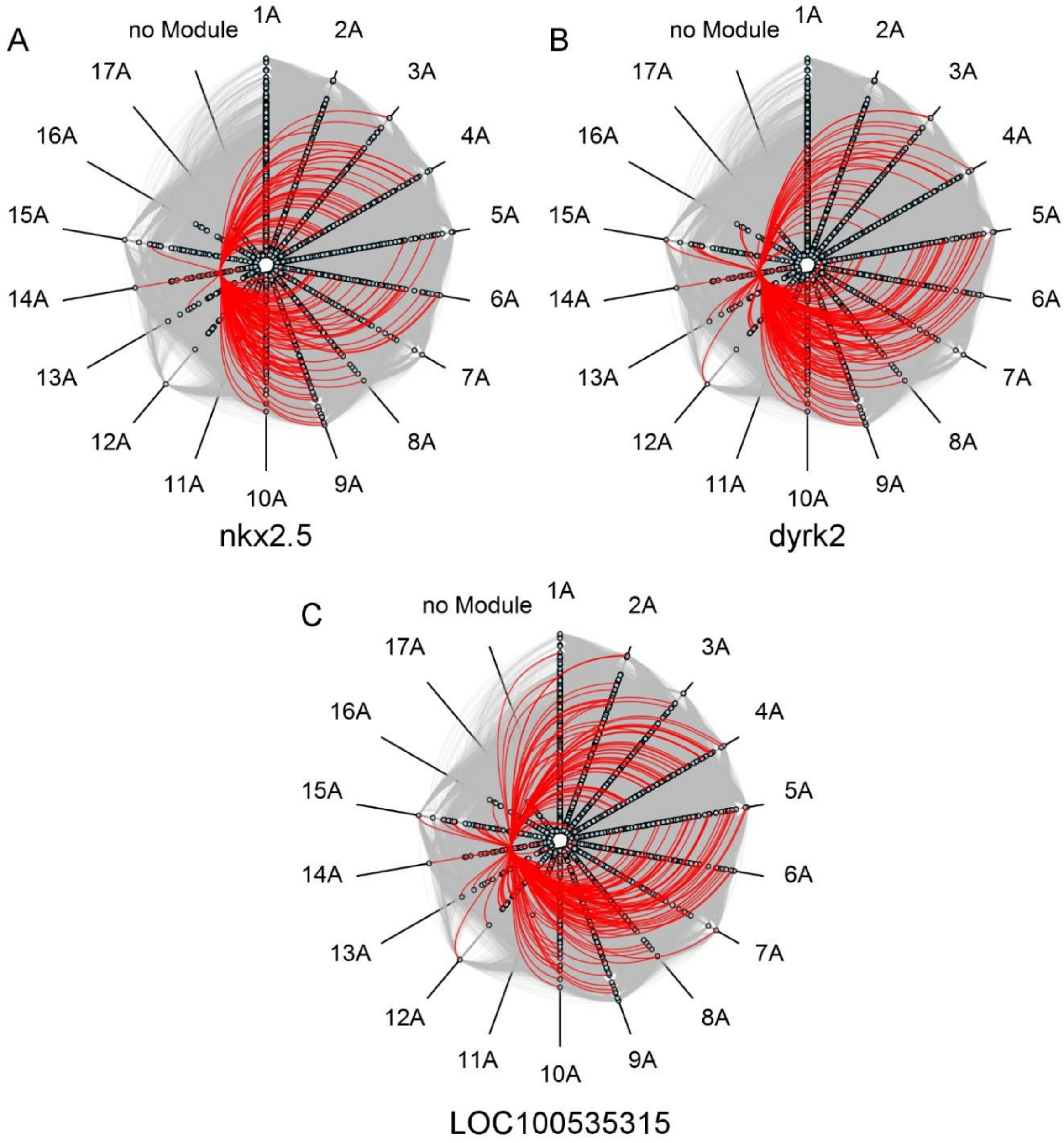

Figure S4. Examining modularity and connectivity patterns of the zebrafish heart regeneration network. Between-module connectivity is graphically depicted (WGCNA modules only), and examples of functionally important or novel genes in module 14A are highlighted: *nkx2.5* **(A)**, *dyrk2* **(B)** and *LOC100535315* **(C)**. Connections involving these genes are highlighted in red, other gene-gene associations are drawn in grey.

Table S2. Network Hubs are Enriched in Ortholog Genes in Human, Rat and Mouse, Related to Results (Network Hubs Are Relevant to Heart Regeneration in Mammals). Proportion of orthologs found for hubs vs. non-hubs in the three organisms independently. All differences in proportions are statistically detectable ($P \leq 0.02$, Chi-square test).

	**Human**		**Rat**		**Mouse**	
	Mapped	Not mapped	Mapped	Not mapped	Mapped	Not mapped
**Hubs**	331	94	332	93	335	90
**Non-hubs**	2149	819	2064	904	2067	901

Supplementary Methods

Zebrafish husbandry and cryoinjury experiments

Experiments were conducted with adult zebrafish between 10 and 11 months old. We used the TU strain (ZIRC, Eugene, OR, USA). Animals were raised at a density of 3 fish/L under standard laboratory conditions in the ZebTEC Stand Alone system (Tecniplast®). All procedures were approved by the Animal Welfare Structure of Luxembourg. Cryoinjury was performed as previously described (Gonzalez-Rosa and Mercader, 2012). Animals were sacrificed at different time points after surgery, from 4 hours to 90 days, by immersion in 0.16% tricaine (Sigma Aldrich, Bornem, BE). Hearts were dissected in PBS containing 2U/mL heparin and 0.1M KCl. Heart ventricles were then used for RNA extraction, and whole hearts were used for histological staining. Heart ventricles or whole hearts of uninjured fish served as controls.

Histological staining and immunohistochemistry

Hearts were immediately fixed overnight at 4°C in 4% PFA, dehydrated, embedded in paraffin wax and cut in 7µm thin sections. Sections were further deparaffinized in xylol, rehydrated in ethanol and washed in distilled water. Connective tissue was visualized using the Masson-Goldner's trichrome staining kit from Merck (Darmstadt, GE), according to the manufacturer's instructions. Hearts were immunostained for tropomyosin or processed for TUNEL staining as previously described (Rodius et al., 2014). Fluorescent pictures were acquired with a confocal fluorescence microscope (Zeiss Laser Scanning Microscope LSM 510, LSM 510 Meta software). Brightfield images were taken with a Leica DMI6000B microscope coupled to a Leica DFC320 camera and processed with the LAS software. Adobe Photoshop® CS6 software was used for image editing.

Transcriptome profiling assays

For each time point and control, five heart ventricles per biological replicate were pooled in TRIzol® (Invitrogen, Carlsbad, CA). Extraction was performed as previously described (Rodius et al., 2014). RNA purity and integrity were monitored using a NanoDrop® ND-1000 spectrophotometer (Thermo Scientific, Wilmington, USA) and Agilent 2100 Bioanalyzer (Agilent Technologies, Paolo Alto, CA). RNAs used in the present study were of good quality and un-degraded (Ratio A260/A280 ≈ 2 and RIN ≥ 7). Transcriptome profiling assays were performed using Zebrafish GeneChip 1.0 ST arrays (Affymetrix, Santa Clara, CA). Briefly, 250 ng of total RNAs were reverse transcribed into cDNA, then transcribed into cRNAs and labeled into biotinylated cRNA using the GeneChip WT Reagent kit (Affymetrix) according to the manufacturer's standard protocols (P/N 4425209 Rev.B and P/N 702808 Rev.6). Labeled products were randomly fragmented and hybridized onto Affymetrix GeneChips. Upon hybridization, arrays were washed and stained using the Affymetrix GeneChip WT Terminal Labeling and Hybridization kit, before being scanned using a GeneChip Scanner 3000 according to the manufacturer's standard procedure (P/N 702731 Rev 3).

Microarray data are available at the NCBI's Gene Expression Omnibus database (GEO Accession Number: GSE67665).

Gene expression data analysis

Microarray data were first pre-processed by Partek® Genomics Suite version 6.5 using the robust multi-chip analysis (RMA) (Irizarry et al., 2003) with GC-content correction. Only features with log2 signal intensity exceeding 6 in at least one sample were kept for further analysis. Here we used empirical Bayes method from *limma* package of R/Bioconductor for differential expression analysis as in (Nazarov et al., 2013). The same *limma* model with specific contrasts was used afterwards to compare expression between time points. Benjamini-Hochberg's false-discovery rate (FDR) was used to adjust p-values for multiple testing. Visualization of the sample clustering and expression patterns among significant genes was performed by PCA plots and heatmaps using a standard functions of R: *prcomp* and *heatmap* concordantly. Lists of differentially expressed genes at each time (vs. control samples) were analysed with DAVID to identify functional enrichments (functional clustering with default parameters) (Huang et al., 2009;). A threshold of FDR < 5% and abs(FoldChange) > 1.6 were applied. The following lists of differentially expressed genes were used as inputs: 2438 genes (at 4h after injury), 2576 (day-1), 2403 (day-3), 1448 (day-7), 947 (day-14) and 899 (day-90) genes. The reference background was *Danio rerio*. To facilitate the visualization of significant functional enrichments in the different sets of differentially expressed genes (Figure 3B and Table S1), we focused on enrichments with DAVID's score > 2 and the resulting associations were curated by a human expert (author S.R.).

Gene co-expression network generation and analysis

Preprocessed microarray data were filtered by variance paired with a q-value false discovery rate method (Hackstadt and Hess, 2009) using multtest (Pollard et al., 2004), genefilter (Gentleman et al., 2009) and qvalue (Storey and Tibshirani, 2003) R packages. To construct the weighted gene co-expression network, we applied the

WGCNA (Langfelder and Horvath, 2008; Zhang and Horvath, 2005). Briefly, we determined co-expression similarity between the probe sets by Pearson's correlation coefficient and converted the correlation matrix into an (unsigned) weighted adjacency matrix with soft threshold β=6. This threshold resulted in a network that exhibited a good balance between useful topological criteria: scale-free fit, median connectivity values and modularity. Also we analyzed other key topological properties such as density, centralization and heterogeneity. To facilitate visualization, reduce potential spurious correlations and focus on most significant associations, we filtered out edges with weights below 0.26, corresponding to Pearson's correlation lower than 0.8 (Borate et al., 2009). The network was visualized in Cytoscape (Shannon et al., 2003), where an edge weight denotes (gene-gene) co-expression and node color corresponds to the differential gene expression at different times (in relation to control samples). Movie was created with Adobe Photoshop® CS6 software.

Modules were detected with WGCNA (Langfelder and Horvath, 2008) and ClusterONE (Nepusz et al., 2012). In WGCNA, an advanced dynamic tree cut technique was implemented with minimum module size = 30 and deepSplit = 2. ClusterONE (under Cytoscape) was applied with default settings (Nepusz et al., 2012), and we focused on statistically significant modules. We estimated overlaps between the modules generated by the two methods with the Jaccard similarity index (Figure S2).

Functional and pathway enrichment analysis of modules was implemented with the Integrative Multi-species Prediction system (IMP) (Wong et al., 2012). IMP predicts functional associations for a given gene set through the integration of high-throughput datasets. IMP identifies overrepresented pathways and functions among genes of the module and predicted associated genes using annotations from the Gene Ontology (Ashburner et al., 2000), MetaCyc (Caspi et al., 2008), KEGG (Kotera et al., 2012) and Reactome (Matthews et al., 2009) databases. For functional/pathway enrichment analysis we focused on *Danio rerio* and set to maximum the number of predicted associated genes.

We applied WiPer (Azuaje, 2014) to identify hub genes based on their (weighted) connectivity scores. Genes with statistically detectable connectivity (adjusted-P < 0.05) were defined as hubs. P-values were estimated with 100e+3 random permutations followed by Bonferroni correction (Azuaje, 2014).

Gene orthology analysis in mammals

The zebrafish symbols were mapped to human, mouse and rat NCBI gene IDs using four different methods: retrieval of homolog gene IDs from The Zebrafish Model Organism Database (ZFIN) (Bradford et al., 2011) or GeneCards (Rebhan et al., 1997), Homologene searches (Wheeler et al., 2003), and BLAST searches for those genes without hits in the aforementioned databases (McGinnis and Madden, 2004). BLAST searches for genes with database hits were also analyzed to verify mapping accuracy. Gene IDs were retrieved from ZFIN and GeneCards by querying with the symbol on these websites and scraping the webpage for homologs in human, mouse and rat. ZFIN was also scraped for NCBI gene and protein IDs in zebrafish. Homologene was searched with the zebrafish gene ID and scraped for homologs in mammals. Zebrafish protein IDs were converted to gene IDs by searching the nuccore database (Wheeler et al., 2003) with the protein ID and scraping the webpage for the GeneID. For uncharacterized zebrafish genes (LOC symbols), GeneIDs were used as queries in BLAST searches against human, mouse and rat genomes. The top hit was considered as a homolog if e-value < 0.05 (after Bonferroni correction for multiple testing). Analyses were implemented in Python. The Chi-square statistical test was applied to estimate the significance of the proportion of mapped hub genes in the three mammals independently.

Hubs as miR targets in zebrafish and mammals

We investigated the involvement of miRNAs in the regulation of the hubs previously identified by querying the miRTarBase resource (Hsu et al., 2008), a experimentally-validated microRNA-target interactions database. In addition, we harvested predicted

microRNA-target interactions by querying the miRNAMap database (Hsu et al., 2008), which includes predictions obtained by three different algorithms, namely, TargetScan (80), miRanda (Enright et al., 2003) and RNAhybrid (Rehmsmeir et al., 2004). Resulting from this analysis we collected 5 and 1102 experimentally validated and predicted interactions, respectively, targeting hub's transcripts in zebra fish. We also explored the possible regulation of the hubs by miRNAs with a known role in cardiac regeneration, angiogenesis, fibrosis and apoptosis in other species (human, mouse and rat). To this end, we used the same resources described above to collect regulatory interactions between a list of 43 selected miRNAs and orthologs of the hub genes.

Data visualization

The circular plots of module connectivity shown in Figure 4 were created using the D3 graphics library for javascript (http://www.d3js.org). The D3 library was also employed to create the hive plots (Figure S4). The latter are based on concepts introduced by Krzywinski *et al.*(2012). Visualizations of the network of hub genes were created with a combination of custom scripts and publicly available software. The visualizations of miR-hub interactions (Figure 6) were created using the RAW online visualization tool (http://raw.densitydesign.org/).

Supplementary references

Ashburner, M. et al. (2000). Gene ontology: tool for the unification of biology. The Gene Ontology Consortium. Nat. Genet. *25*, 25–9.

Caspi, R. et al. (2008). The MetaCyc Database of metabolic pathways and enzymes and the BioCyc collection of Pathway/Genome Databases. Nucleic Acids Res. *36*, D623–31.

Culhane, A.C., Thioulouse, J., Perriere, G., and Higgins, D.G. (2005). MADE4: an R package for multivariate analysis of gene expression data. Bioinformatics *21*, 2789-2790.

Krzywinski, M., Birol, I., Jones, S. J. M. & Marra, M. A. (2012). Hive plots--rational approach to visualizing networks. Brief. Bioinform. 13, 627–44.

Kotera, M., Hirakawa, M., Tokimatsu, T., Goto, S. & Kanehisa, M. (2012). The KEGG databases and tools facilitating omics analysis: latest developments involving human diseases and pharmaceuticals. Methods Mol. Biol. *802*, 19–39.

Matthews, L. et al. (2009). Reactome knowledgebase of human biological pathways and processes. Nucleic Acids Res. *37*, D619–22.

Wong, A. K. *et al.* (2012) IMP: a multi-species functional genomics portal for integration, visualization and prediction of protein functions and networks. *Nucleic Acids Res. 40*, W484–90.

Zhang, B., and Horvath, S. (2005). A general framework for weighted gene co-expression network analysis. Stat Appl Genet Mol Biol 4, Article17.

Video legend

Movie S1. Animation of Gene Expression Changes in the Regeneration Network, Related to Figure 3 and Figure S1. Global view of changes in gene expression (fold-changes in relation to controls) is provided for each heart regeneration time point. Red nodes: Up-regulated, blue: Down-regulated, white: no change.
