# Supplementary figures and images for: Analysis of the dynamic co-expression network of heart regeneration in the zebrafish
Source: Sci Rep. 2016 May 31;6:26822. doi: 10.1038/srep26822 (PMC4886216; doi:10.1038/srep26822)

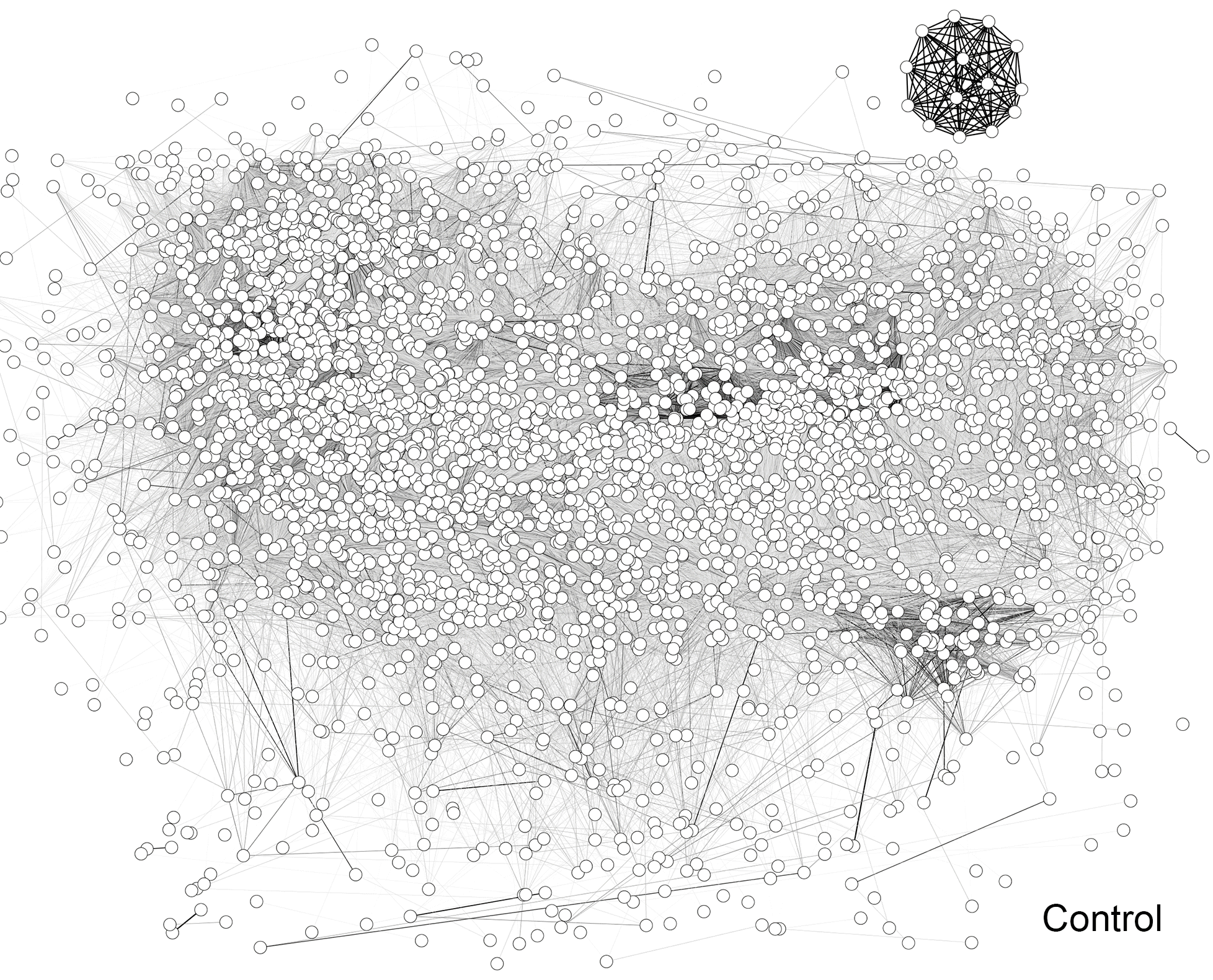

Supplement: Supplementary file 3 — Supplementary Movie S1 (GIF 5561 kb) [file 41598_2016_BFsrep26822_MOESM19_ESM.gif]
